# Supplementary material for: Severe and widespread coral reef damage during the 2014-2017 Global Coral Bleaching Event
Source: Nat Commun. 2026 Feb 10;17:1318. doi: 10.1038/s41467-025-67506-w (PMC12891614; doi:10.1038/s41467-025-67506-w)
Supplement: Supplementary file 1 — Supplementary Information [file 41467_2025_67506_MOESM1_ESM.pdf]

Severe and widespread coral reef damage during the 2014-2017 Global Coral Bleaching Event

Supplementary Tables and Figures

Supplementary Table 1. Newly implemented NOAA Coral Reef Watch coral bleaching heat stress category system for its Bleaching Alert Area product (December 15, 2023).

Stress Level	Definition	Potential Bleaching and Mortality
No Stress	HotSpot ≤ 0°C	No Bleaching
Bleach Watch	0 < HotSpot < 1°C	
Bleaching Warning	1 ≤ HotSpot and 0 < DHW < 4°C-weeks	Risk of Possible Bleaching
Bleaching Alert Level 1	1 ≤ HotSpot and 4 ≤ DHW < 8°C-weeks	Risk of Reef-Wide Bleaching
Bleaching Alert Level 2	1 ≤ HotSpot and 8 ≤ DHW < 12°C-weeks	Risk of Reef-Wide Bleaching with Mortality of Heat-Sensitive Corals
Bleaching Alert Level 3	1 ≤ HotSpot and 12 ≤ DHW < 16°C-weeks	Risk of Multi-Species Mortality
Bleaching Alert Level 4	1 ≤ HotSpot and 16 ≤ DHW < 20°C-weeks	Risk of Severe, Multi-Species Mortality (> 50% of corals)
Bleaching Alert Level 5	1 ≤ HotSpot and DHW ≥ 20°C-weeks	Risk of Near-Complete Mortality (> 80% of corals)

a)

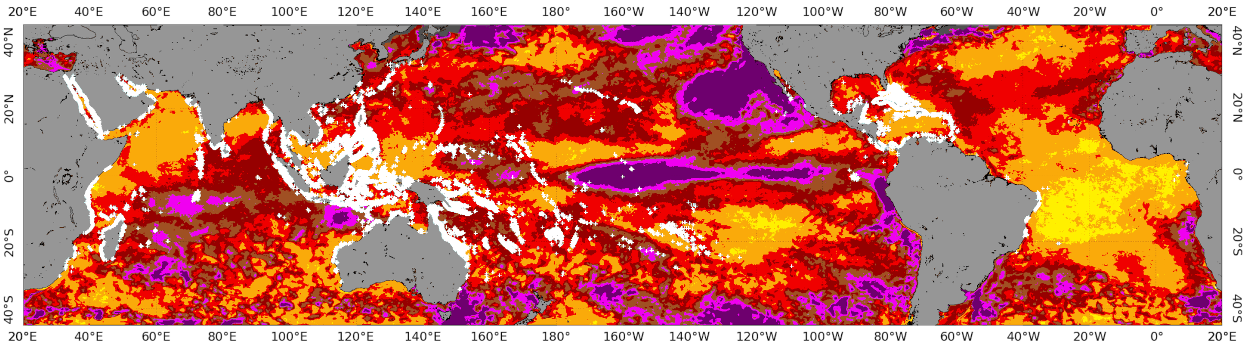
20°E
40°E
60°E
80°E
100°E
120°E
140°E
160°E
180°
160°W
140°W
120°W
100°W
80°W
60°W
40°W
20°W
0°
40°N
20°N
0°
20°S
40°S

b)

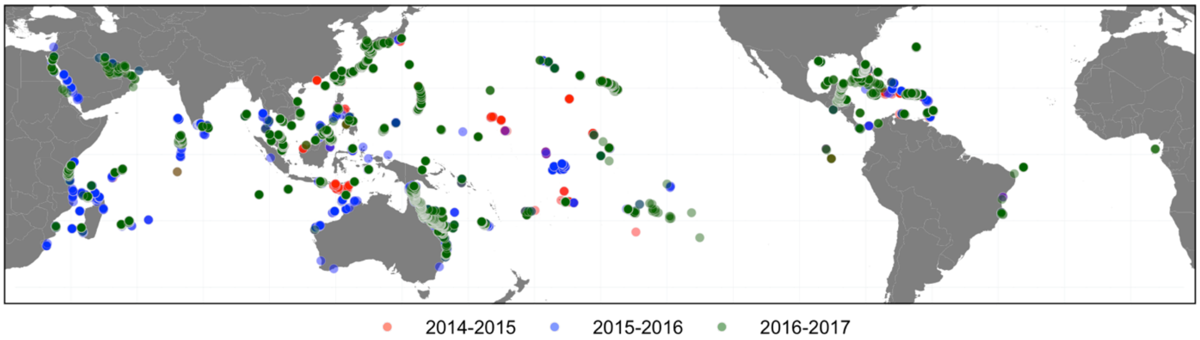
2014-2015
2015-2016
2016-2017

c)

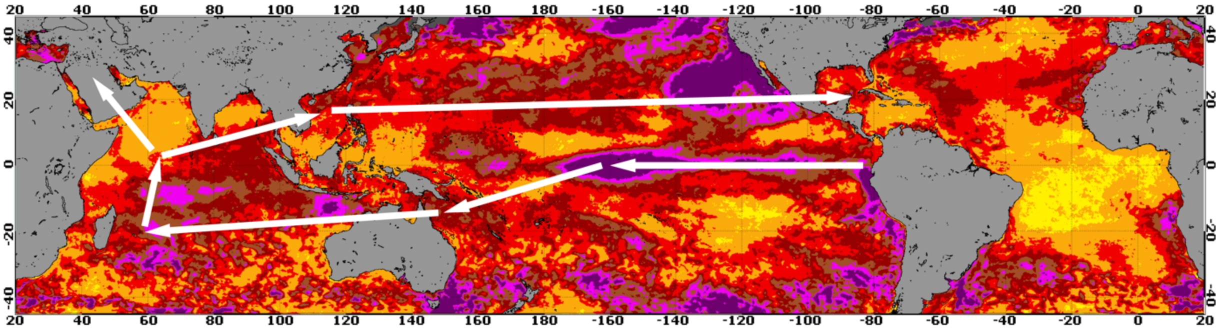

d)

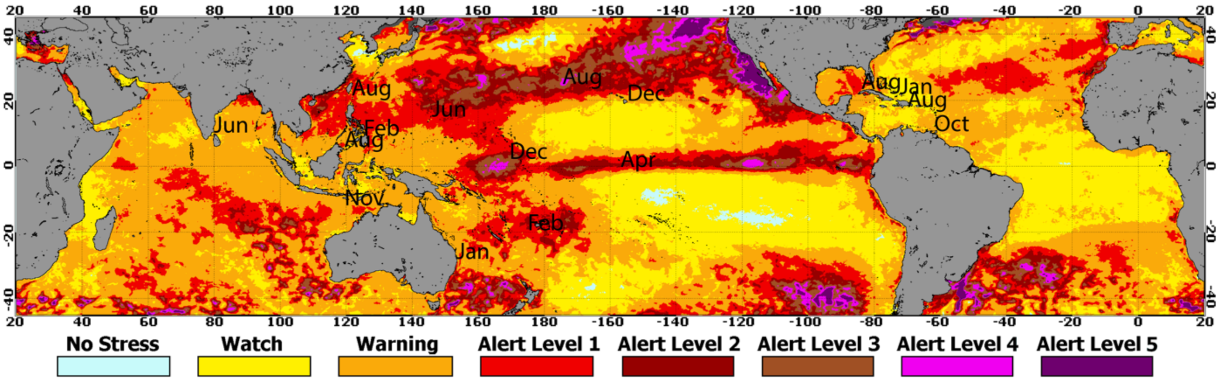
No Stress
Watch
Warning
Alert Level 1
Alert Level 2
Alert Level 3
Alert Level 4
Alert Level 5

e)

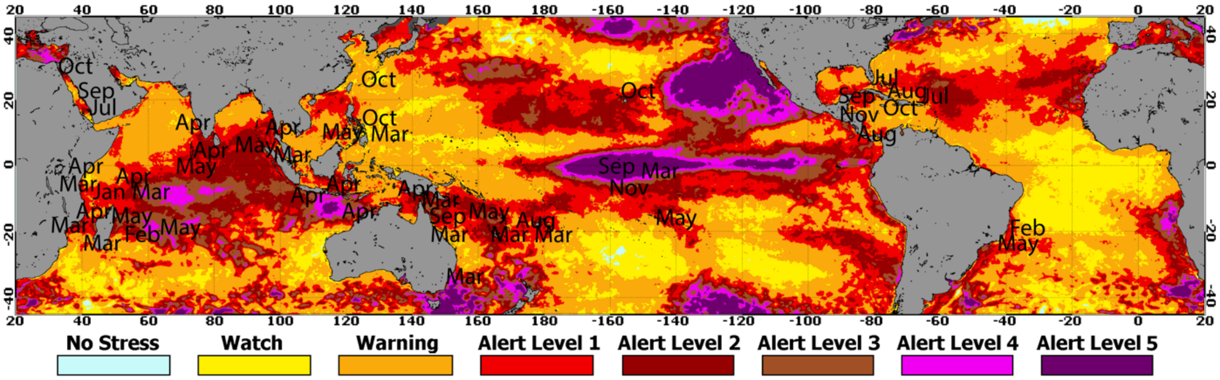
No Stress
Watch
Warning
Alert Level 1
Alert Level 2
Alert Level 3
Alert Level 4
Alert Level 5

f)

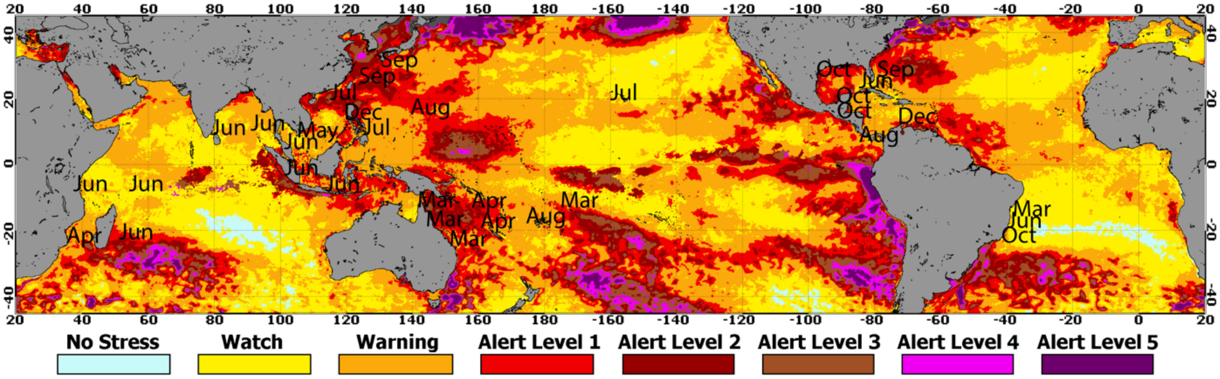
No Stress
Watch
Warning
Alert Level 1
Alert Level 2
Alert Level 3
Alert Level 4
Alert Level 5

Supplementary Figure 1. a Global spatial pattern of maximum heat stress from 2014–17. Heat stress categories of Alert Levels 1 and 2 correspond to moderate reef-wide coral bleaching ($4 \leq DHW < 8$°C-weeks) and severe reef-wide bleaching with moderate mortality ($8 \leq DHW < 12$°C-weeks), respectively. Newly established Alert Levels 3–5 correspond to risk of increasingly severe mortality of corals across reefs. White pluses plotted over underlying heat stress denote pixels containing coral reefs. **b** Map of contributed field surveys of bleaching or mortality in each bleaching year. Overlapping survey dots result in blending of colors, with gray colors denoting sampling in all three years. **c** White arrows show the typical, generalized sequence of heat stress and bleaching during global coral bleaching events (similar patterns were seen in GCBE1 and GCBE2, and **d-f** show the sequence of maximum heat stress and bleaching during each bleaching year during GCBE3. Black text on the Bleaching Alert Level maps for each bleaching year are the months in which bleaching severity of 50% or higher was first reported (bleaching data found in Supplementary Data 1). The data and code to generate this figure are provided in Zenodo (https://doi.org/10.5281/zenodo.15114357).

Supplementary Table 2. Percentage and numbers of ~5×5 km^2 reef-containing pixels that reached or exceeded CRW's Bleaching Alert Levels 1–5 for each of the three bleaching years during GCBE3 as well occurring two or more times during 2014–17. All percentages are relative to the total number of reef pixels in water (n=53,997); values are cumulative (i.e., pixels that reached Alert Level 5 are also counted in all lower Alert Levels). The data and code to generate this table are provided in Zenodo (https://doi.org/10.5281/zenodo.15114357).

	Percent (number) of pixels reaching or exceeding each CRW Bleaching Alert Level				
	Alert Level 1 (DHW ≥ 4°C-weeks)	Alert Level 2 (DHW ≥ 8°C-weeks)	Alert Level 3 (DHW ≥ 12°C-weeks)	Alert Level 4 (DHW ≥ 16°C-weeks)	Alert Level 5 (DHW ≥ 20°C-weeks)
2014–15	18.1% (9765)	2.3% (1215)	0.4% (202)	0.1% (74)	<0.1% (15)
2015–16	47.7% (25759)	14.8% (7978)	3.0% (1639)	0.6% (300)	0.1% (60)
2016–17	36.8% (19855)	11.8% (6361)	2.5% (1357)	0.1% (80)	<0.1% (22)
2014–17	65.8% (35528)	23.6% (12751)	5.0% (2721)	0.7% (394)	0.1% (75)
Multiple Events	31.1% (16768)	4.9% (2655)	0.9% (468)	0.1% (60)	<0.1% (22)

a)

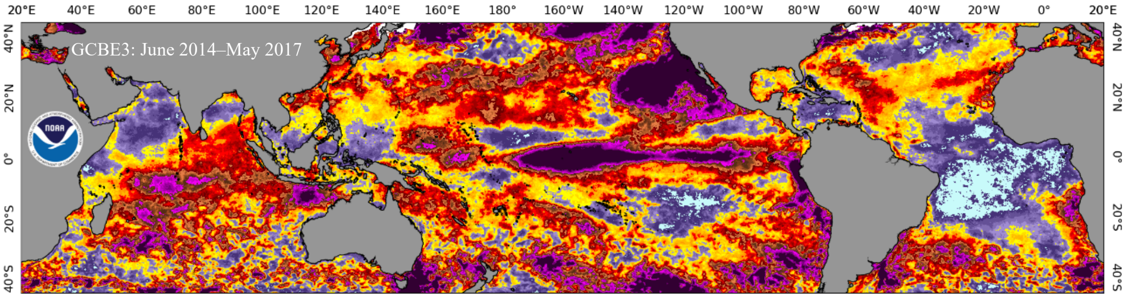

b)

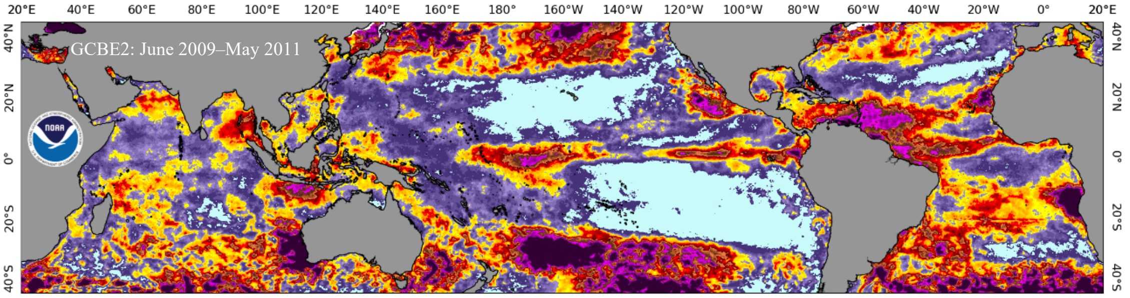

c)

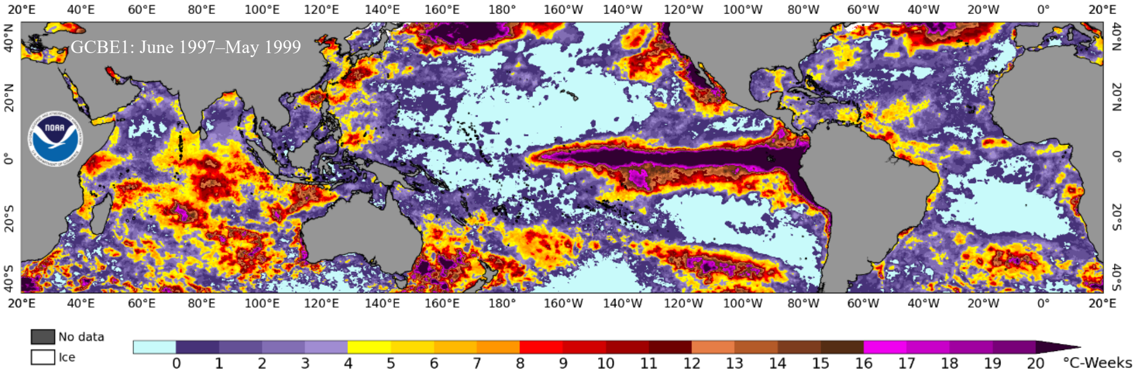

Supplementary Figure 2. Global pattern of maximum Degree Heating Week (DHW) from **a** the Third Global Coral Bleaching Event (GCBE3) 2014–17, **b** GCBE2 2009–2011, and **c** GCBE1 1997–1999. Moderate reef-wide coral bleaching has been linked to $4 \leq$ DHW < 8°C-weeks, and severe reef-wide bleaching with moderate mortality to $8 \leq$ DHW < 12°C-weeks. See Supplementary Fig. 1a for global coral reef locations. The data and code to generate this figure are provided in Zenodo (https://doi.org/10.5281/zenodo.15114357).

Supplementary Table 3. Predicted impacts during the GCBE3. Sums of ~5×5 km^2 satellite pixel values within the 21 GCBE3 regions depicted in Fig.4. Left two tables provide sum of pixel probabilities for bleaching and mortality in each bleaching year. Third table from left shows percentage of three-year sum of bleaching pixels predicted to also contain moderate or greater mortality (>10% of corals). Fourth table (far right) shows total number of pixels in each GCBE3 region. GCBE3 region #22 (Eastern Atlantic) not shown on Fig. 4 due to small reef area (4 of 53,997 reef-containing pixels). Source data for predicting bleaching and mortality as a function of heat stress are in Supplementary Data 1. Additional data and code to generate this table are provided in Zenodo (https://doi.org/10.5281/zenodo.15114357).

Sum pixel-probability of moderate bleaching

	2014-2015	2015-2016	2016-2017	
1	773.71	1814.03	1443.48	Middle East
2	430.07	1832.7	1753.74	Eastern Indian Ocean
3	456.51	1677.9	1692.21	Central Indian Ocean
4	257.97	983.5	527.07	Southeast Asia
5	249.72	216.54	424.18	East Asia
6	971.28	4352.72	3083.24	Coral Triangle
7	316.29	188.44	947.37	Micronesia
8	239.12	328.1	9.66	Hawaii
9	1060.34	1991.13	912.05	Florida Straits
10	82.39	118.31	89.56	Gulf of Mexico
11	329.13	1033.08	619.45	Western Caribbean
12	213.76	942.98	304.6	Northern Caribbean
13	64.04	470.23	370.48	Eastern Caribbean
14	98.52	175.17	113.29	Eastern Pacific
15	146.95	1916.88	1696.65	Western Indian Ocean
16	125.1	455.22	323.88	Western Australia
17	1489.85	4289.46	1743.62	Melanesia
18	271.77	2618.9	1836.84	GBR
19	222.12	945.8	573.84	Polynesia
20	203.79	324.57	81.12	Kiribati
21	83.25	207.19	141.7	Brazil
22	0.16	0	0.75	Eastern Atlantic

Sum pixel-probability of moderate mortality

	2014-2015	2015-2016	2016-2017	
1	304.62	664.1	590.3	Middle East
2	195.61	738.06	677.9	Eastern Indian Ocean
3	177.59	846.21	810.03	Central Indian Ocean
4	182.16	248.58	265.65	Southeast Asia
5	113.17	61.5	163.42	East Asia
6	808.93	1247.03	1473.46	Coral Triangle
7	182.87	73.51	376.34	Micronesia
8	92.46	71.71	8.92	Hawaii
9	102.7	122.25	129.48	Florida Straits
10	6.76	4.68	4.14	Gulf of Mexico
11	75.54	56.43	62.41	Western Caribbean
12	42.08	75.26	68.73	Northern Caribbean
13	22.6	36.99	32.5	Eastern Caribbean
14	10.18	7.63	7.67	Eastern Pacific
15	74.6	757.59	522.43	Western Indian Ocean
16	80.99	148.37	130.49	Western Australia
17	760.68	1004.84	844.7	Melanesia
18	334.09	672.22	750.39	GBR
19	88.73	215.94	254.38	Polynesia
20	67.04	106.76	40.7	Kiribati
21	17.44	13.38	12.56	Brazil
22	0.08	0	0.23	Eastern Atlantic

% of bleaching pixels with mortality

	2014-17	
1	39%	Middle East
2	40%	Eastern Indian Ocean
3	48%	Central Indian Ocean
4	39%	Southeast Asia
5	38%	East Asia
6	42%	Coral Triangle
7	44%	Micronesia
8	30%	Hawaii
9	9%	Florida Straits
10	5%	Gulf of Mexico
11	10%	Western Caribbean
12	13%	Northern Caribbean
13	10%	Eastern Caribbean
14	7%	Eastern Pacific
15	36%	Western Indian Ocean
16	40%	Western Australia
17	35%	Melanesia
18	37%	GBR
19	32%	Polynesia
20	35%	Kiribati
21	10%	Brazil
22	34%	Eastern Atlantic

Number of reef pixels in GCBE region

	nReefPix
Middle East	3501
Eastern Indian Ocean	2403
Central Indian Ocean	2053
Southeast Asia	2644
East Asia	1071
Coral Triangle	13803
Micronesia	2009
Hawaii	520
Florida Straits	2740
Gulf of Mexico	135
Western Caribbean	1314
Northern Caribbean	1560
Eastern Caribbean	728
Eastern Pacific	214
Western Indian Ocean	2669
Western Australia	804
Melanesia	8034
GBR	5317
Polynesia	2005
Kiribati	403
Brazil	292
Eastern Atlantic	4

Supplementary Table 4. Model selection for moderate or greater bleaching (>10% of corals). Codes indicate the fixed effect structure of each model. "DHW" indicates Degree-Heating-Weeks (^{0}C-weeks) and is a continuously varying predictor. "R" and "Y" indicate the categorical variables "ocean basin" (Asia-Pacific, Caribbean/Atlantic, or Indian Ocean/Middle East) and "bleaching year" (2014–2015, 2015–2016, or 2016–2017), respectively. "+" indicates an additive effect, "x" indicates an interaction (inclusive of main and interactive effects), ":" indicates an interaction effect only (not including fixed effects), and "-" indicates the removal of a particular effect. For example, DHWxRxY – R:Y indicates a model including all main effects and interactions involving DHW, ocean basin, and bleaching year, except for the interaction between basin and bleaching year. Similarly, DHWxRxY - DHW:Y:R – Y:R indicates a model with all main effects and interactions except for a three-way interaction of DHW, Y, and R, or an interaction between Y and R. "df" is degrees of freedom, "AIC" is Akaike's Information Criterion, and D is the difference between the AIC of the given model and that of the best model in the model set (and so by definition the best-fitting model has D=0). The data and code to generate this table are provided in Zenodo (https://doi.org/10.5281/zenodo.15114357).

**Model**	**df**	**AIC**	**Δ**
DHWxRxY – R:Y	15	12491.0	0
DHWxRxY	19	12495.4	4.4
DHWxY + R	9	12511.4	20.4
DHWxRxY - DHW:Y:R – Y:R	11	12514.3	23.3
DHWxRxY – R:Y – R – Y	11	12518.8	27.8
DHWxRxY – DHW:R:Y	15	12521.1	30.1
DHWxY	7	12540.5	49.4
DHWxR + Y	9	12607.7	116.7
DHWxR	7	12612.0	121.0
DHWxR + R:Y + Y	13	12614.9	123.9
DHW + R + Y	7	12623.7	132.7
DHW + R	5	12628.2	137.2
DHW + RxY	11	12630.6	139.6
DHW + Y	5	12653.7	162.7
DHW	3	12657.9	166.9

Supplementary Table 5. Model selection for severe bleaching (>50% coral bleached). See Supplementary Table 4 for an explanation of model codes and the fixed effect structure of each model.

Model	df	AIC	Δ
DHWxRxY – RxY	11	8109.5.0	0
DHWxRxY – R:Y	15	8112.5	3.0
DHWxRxY	19	8117.1	7.6
DHWxRxY - DHW:Y:R – Y:R	11	8123.1	13.6
DHWxRxY – DHW:R:Y	15	8128.7	19.2
DHWxY + R	9	8135.9	26.4
DHWxY	7	8136.8	27.3
DHWxR + Y	9	8141.3	31.8
DHWxR	7	8141.5	32.0
DHWxR + Y:R + Y	13	8147.0	37.5
DHW + R	5	8174.9	65.4
DHW + R + Y	7	8175.2	65.7
DHW + Y	5	8176.2	66.7
DHW	3	8177.0	67.4
DHW + RxY	11	8180.9	71.4

Supplementary Table 6. Model selection for moderate or greater mortality (>10% of corals)). See Supplementary Table 4 for an explanation of model codes and the fixed effect structure of each model.

**Model**	**df**	**AIC**	**Δ**
DHWxRxY	19	4824.5	0
DHWxRxY – RxY	11	4826.3	1.8
DHWxRxY – R:Y	15	4826.7	2.2
DHWxRxY – DHW:R:Y	15	4842.7	18.2
DHWxY – DHWxR	11	4849.2	24.7
DHWxY + Y:R + Y	13	4878.2	53.7
DHWxR	7	4880.5	56.0
DHWxR + Y	9	4883.2	58.7
DHWxY	7	4907.1	82.6
DHWxY + R	9	4910.7	86.2
DHW	3	4922.6	98.1
DHW + RxY	11	4923.8	99.3
DHW + Y	5	4925.5	101.0
DHW + R	5	4925.9	101.4
DHW + R + Y	7	4929.0	104.5

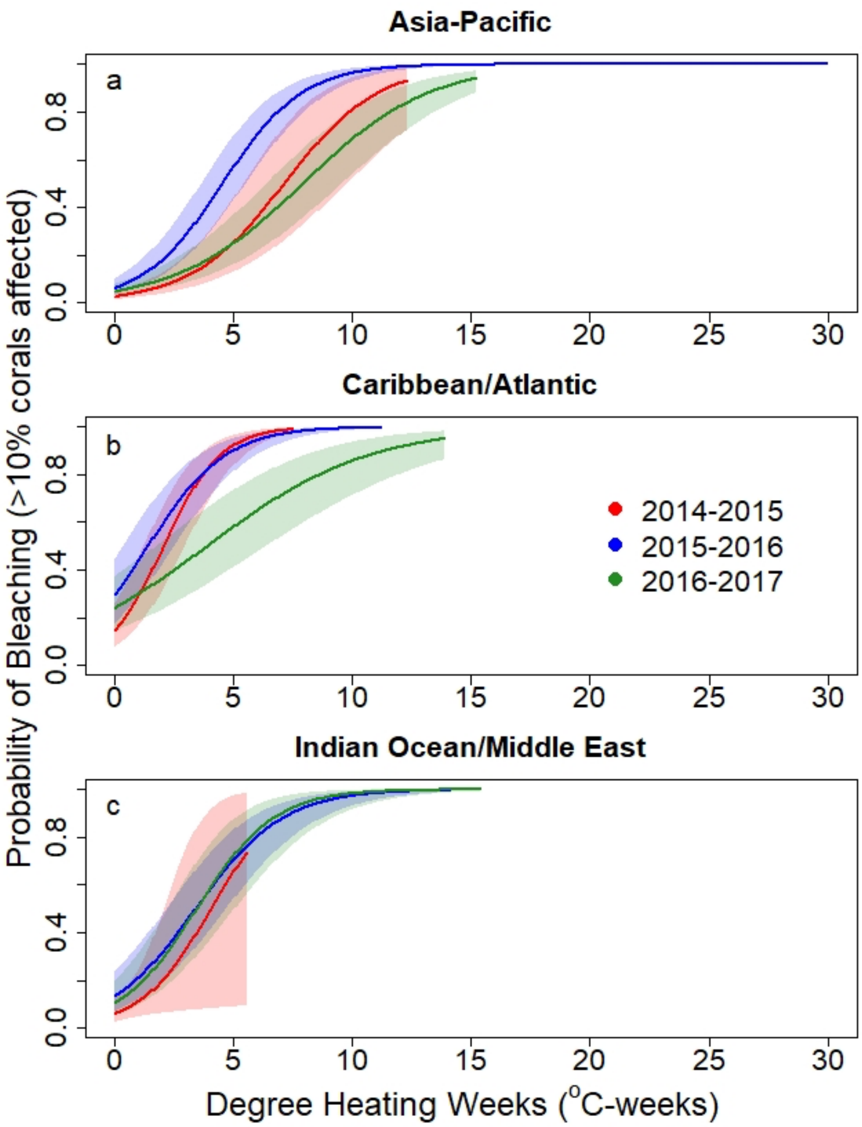

Supplementary Figure 3. The response curves for moderate or greater bleaching (>10% of corals) as a function of heat stress varied among basins and bleaching years. Bleaching response curves, with 95% confidence limits (shading), in each of the three basins in the three bleaching years (2014–15, 2015–16, 2016–17): **a** Asia-Pacific (AP), **b** Caribbean/Atlantic (CA), and **c** Indian Ocean/Middle East (IM). The vertical axis is the probability of moderate or greater bleaching calculated from the bleaching/mortality database (*n* = 8,089 surveys in AP; 5,426 in CA; and 1,587 in IM). Curves extend across the domain of DHW values apparent for that basin and year. Solid lines represent the mean probability of bleaching, and shaded regions represent 95% confidence intervals on the mean. The data and code to generate this figure are provided in Zenodo (https://doi.org/10.5281/zenodo.15114357).

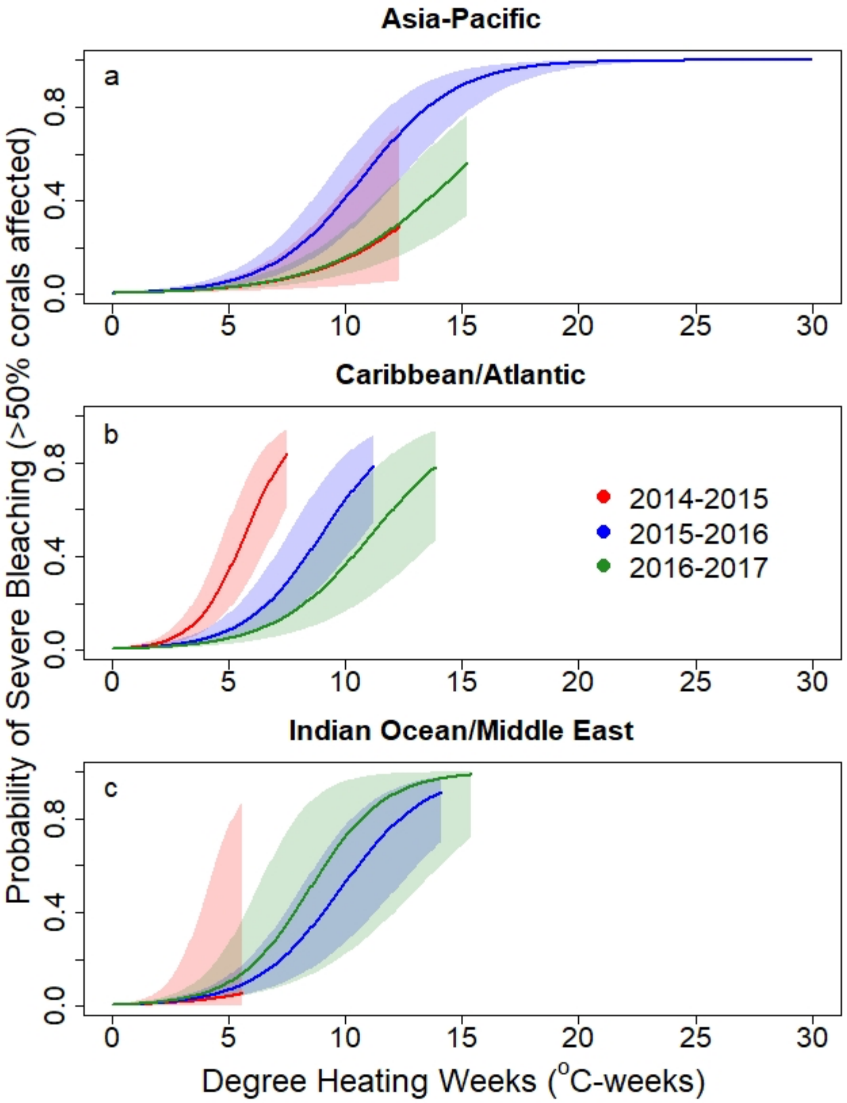

Supplementary Figure 4. The response curves for severe coral bleaching (affecting > 50% of corals) as a function of heat stress varied among basins and bleaching years. Bleaching response curves, with 95% confidence limits (shading), in each of the three basins in the three bleaching years (2014–15, 2015–16, 2016–17): **a** Asia-Pacific (AP), **b** Caribbean/Atlantic (CA), and **c** Indian Ocean/Middle East (IM). The vertical axis is the probability of severe bleaching calculated from the bleaching/mortality database (n = 8,089 surveys in AP; 5,426 in CA; and 1,587 in IM). Curves extend across the domain of DHW values apparent for that basin and year. Solid lines represent the mean probability of bleaching, and shaded regions represent 95% confidence intervals on the mean. The data and code to generate this figure are provided in Zenodo (https://doi.org/10.5281/zenodo.15114357).

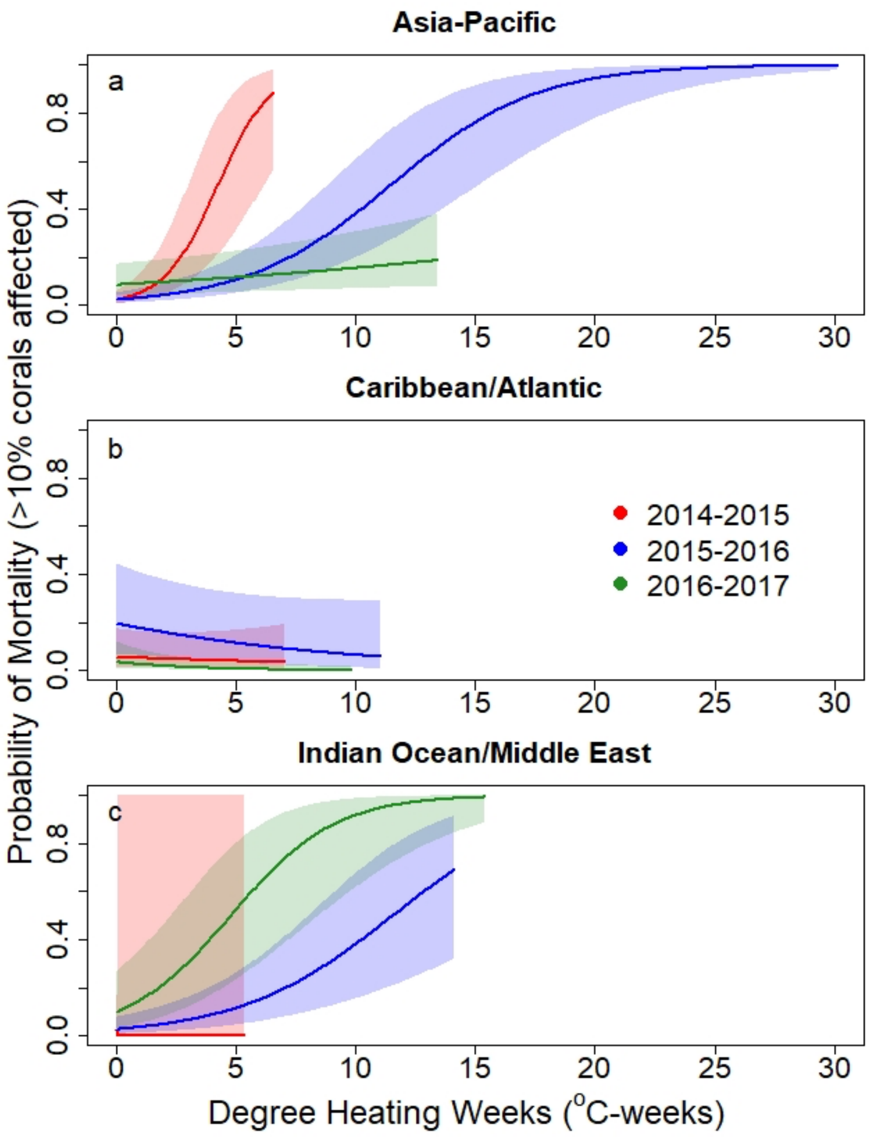

Supplementary Figure 5. The response curves for moderate or greater mortality (>10% of corals) as a function of heat stress varied among basins and bleaching years. Mortality response curves, with 95% confidence limits (shading), in each of the three basins in the three bleaching years (2014–15, 2015–16, 2016–17): **a** Asia-Pacific (AP), **b** Caribbean/Atlantic (CA), and **c** Indian Ocean/Middle East (IM). The vertical axis is the probability of moderate or greater mortality calculated from the bleaching/mortality database (n = 8,089 surveys in AP; 5,426 in CA; and 1,587 in IM). Curves extend across the domain of DHW values apparent for that basin and year. Solid lines represent the mean probability of bleaching, and shaded regions represent 95% confidence intervals on the mean. The data and code to generate this figure are provided in Zenodo (https://doi.org/10.5281/zenodo.15114357).

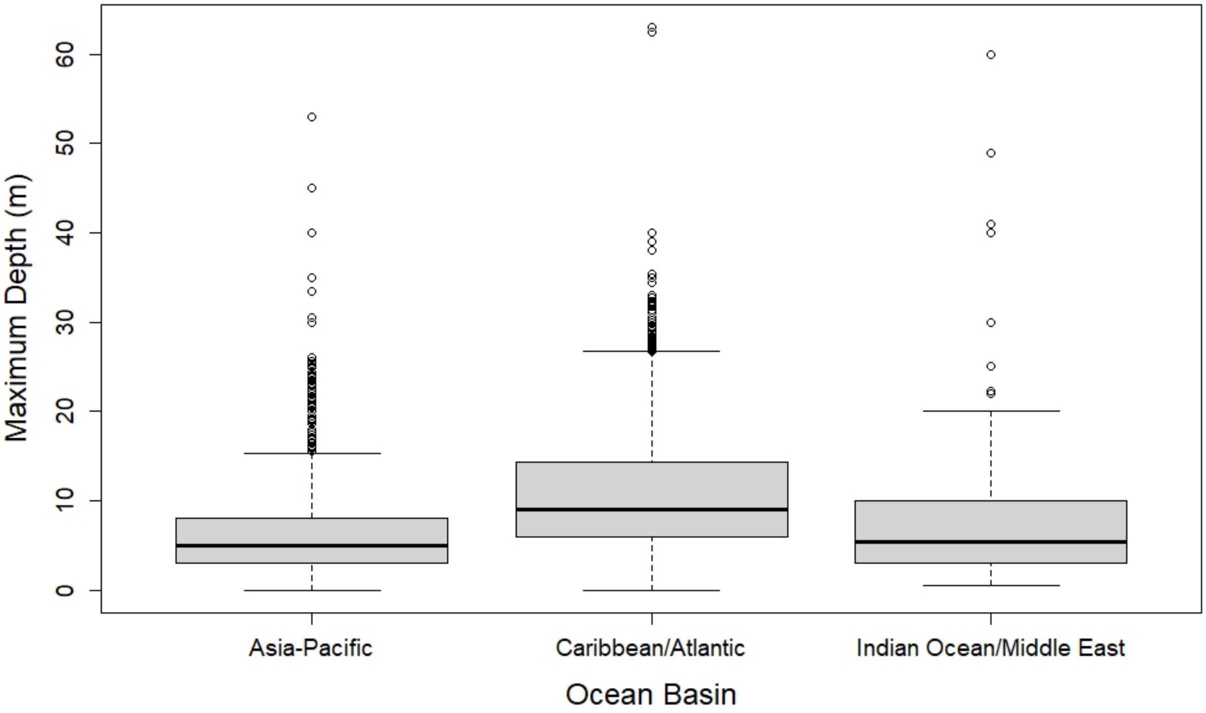

Supplementary Figure 6. Boxplots of observation depths by ocean basin. Note that observations from the Caribbean tend to be somewhat deeper, on average, than those from the other two ocean basins (n = 5,547 surveys in AP; 5,158 in CA; and 1,347 in IM). Boxes represent the inter-quartile range, the thick horizontal line is the median, and the whiskers extend out by 1.5 times the inter-quartile range, or to the most extreme observation, whichever is closer to the median. Points represent individual observations whose values are more extreme than the outer edge of the whisker. The data and code to generate this figure are provided in Zenodo (https://doi.org/10.5281/zenodo.15114357).

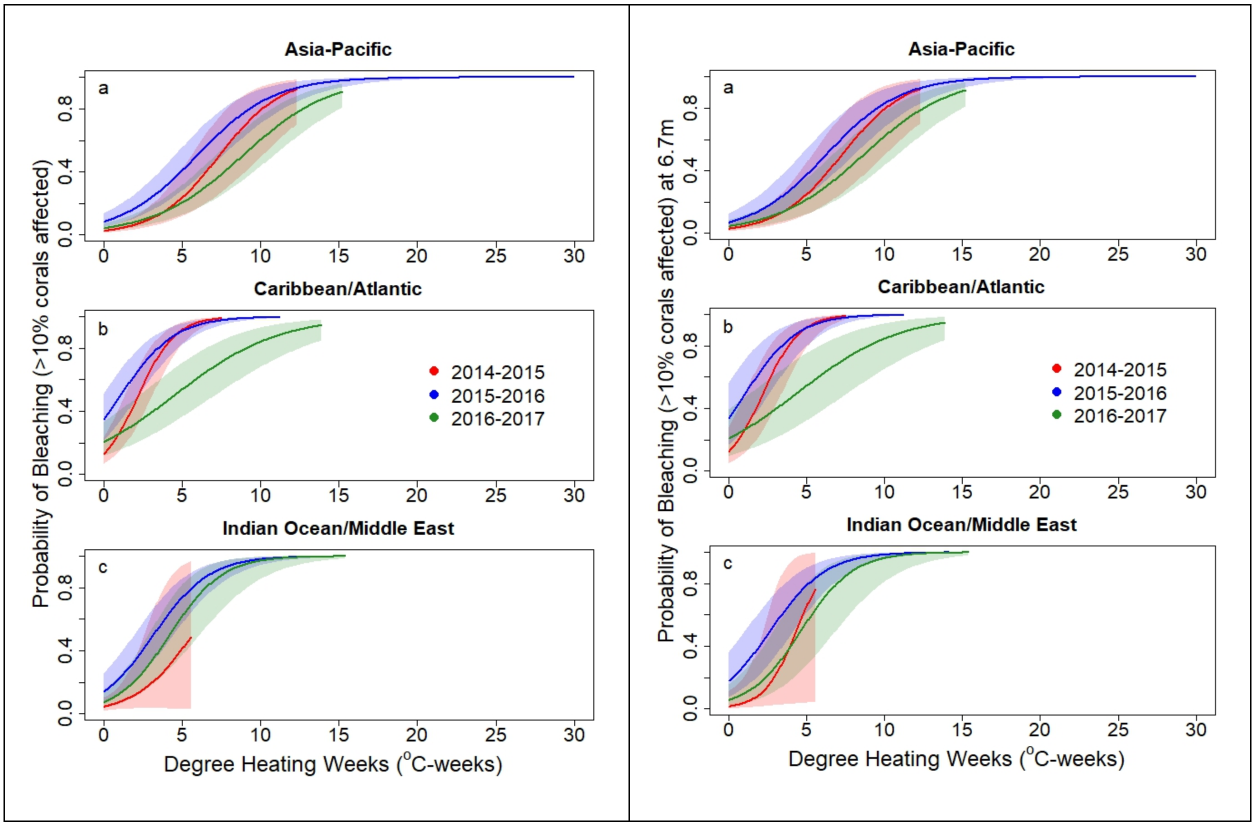

Supplementary Figure 7. Analysis of moderate or greater bleaching (>10% of corals) for the subset of locations with maximum depth of observations recorded. The left panels show the best-fitting model not including depth: the correspondence between these fits and those of the full analysis (Supplementary Fig. 3) indicate the extent to which this subset of observations is representative of the full analysis. The right panels show the best-fitting model including depth as a covariate. For visualization of the basin and interannual variability in bleaching thresholds, these latter thresholds represent predicted bleaching probabilities at the dataset's median depth of 6.7m. The vertical axis is the probability of moderate or greater coral bleaching calculated from the subset of the bleaching/mortality database that contained maximum depth values (n = 5,547 surveys in AP; 5,158 in CA; and 1,347 in IM). Curves extend across the domain of DHW values apparent for that basin and year. Solid lines represent the mean probability of bleaching, and shaded regions represent 95% confidence intervals on the mean. The data and code to generate this figure are provided in Zenodo (https://doi.org/10.5281/zenodo.15114357).

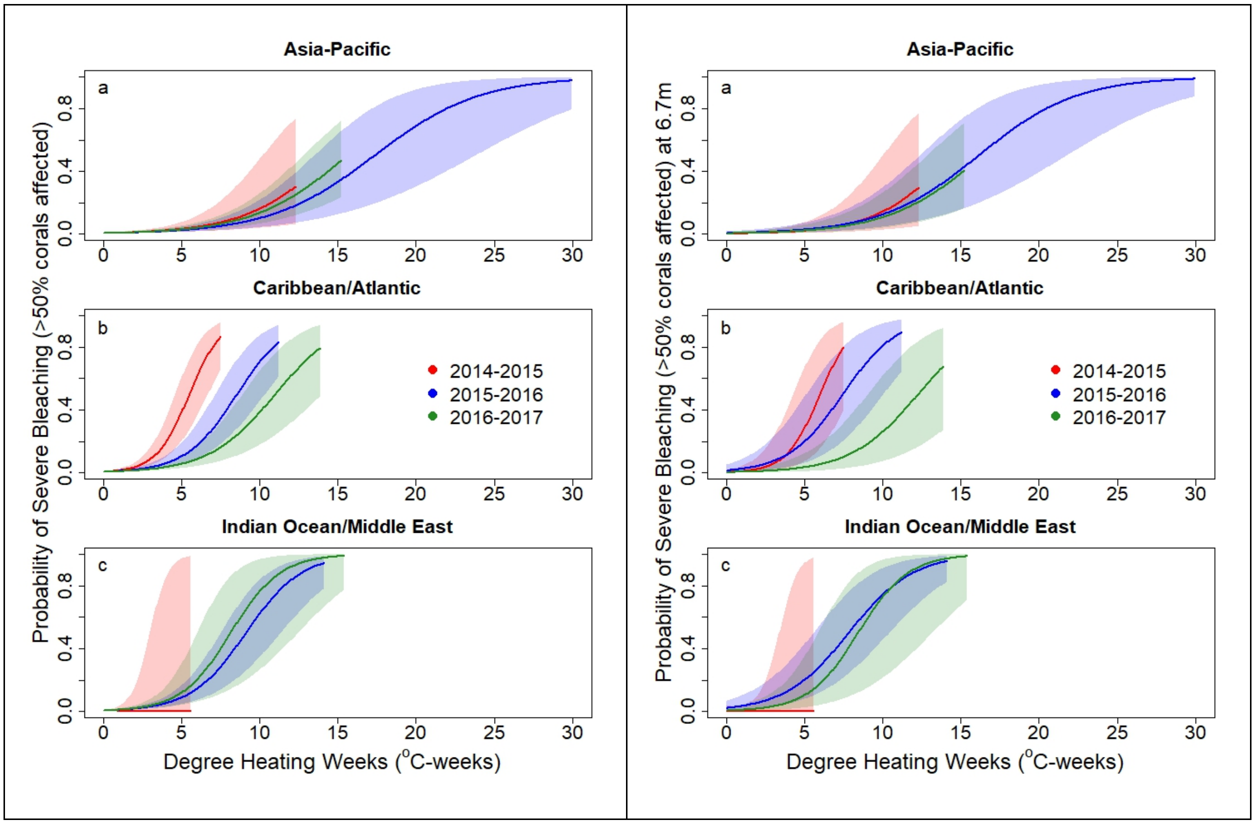

Supplementary Figure 8. Analysis of severe bleaching (affecting >50% of corals) for the subset of locations with maximum depth of observations recorded. The left panels show the best-fitting model not including depth: the correspondence between these fits and those of the full analysis (Supplementary Fig. 4) indicate the extent to which this subset of observations is representative of the full analysis. The right panels show the best-fitting model including depth as a covariate. For visualization of the basin and interannual variability in bleaching thresholds, these latter thresholds represent predicted bleaching probabilities at the dataset's median depth of 6.7m. The vertical axis is the probability of severe coral bleaching calculated from the subset of the bleaching/mortality database that contained maximum depth values (n = 5,547 surveys in AP; 5,158 in CA; and 1,347 in IM). Curves extend across the domain of DHW values apparent for that basin and year. Solid lines represent the mean probability of bleaching, and shaded regions represent 95% confidence intervals on the mean. The data and code to generate this figure are provided in Zenodo (https://doi.org/10.5281/zenodo.15114357).

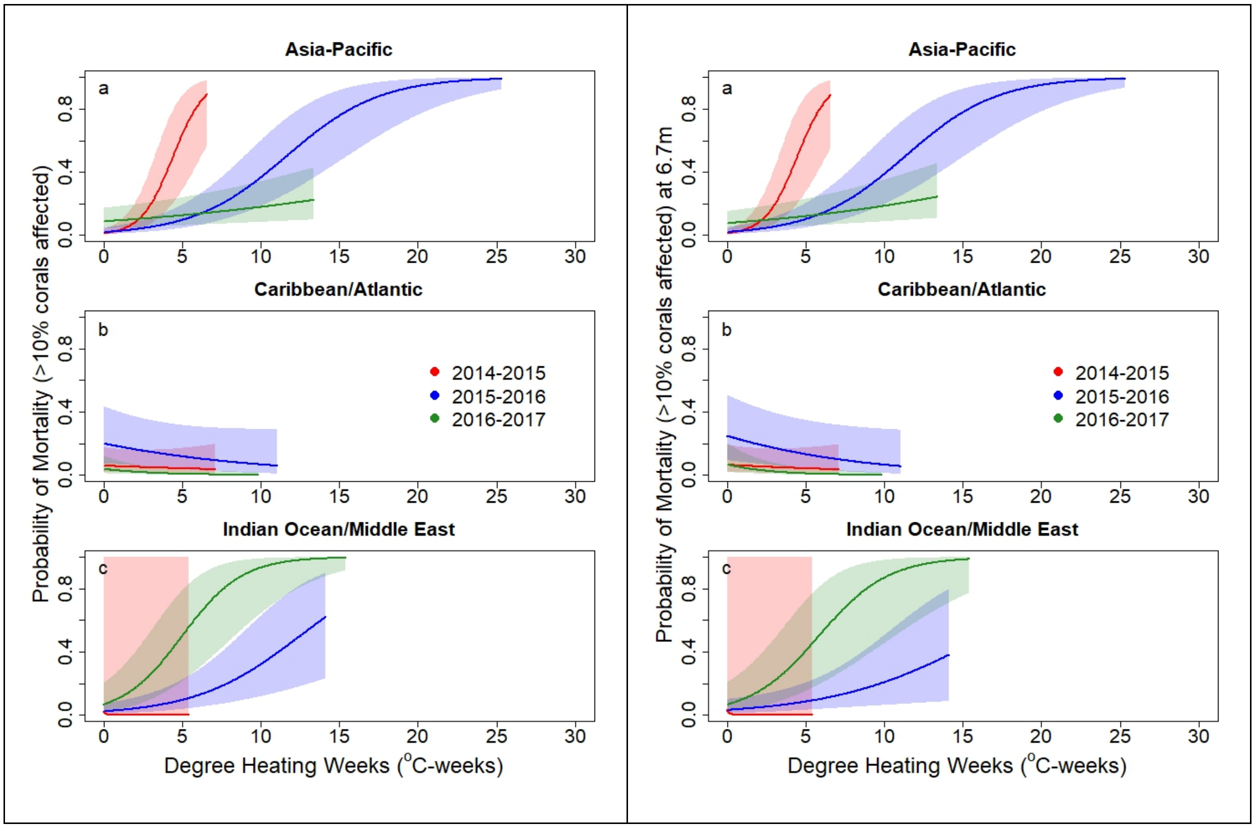

Supplementary Figure 9. Analysis of moderate or greater mortality (>10% of corals) for the subset of locations with maximum depth of observations recorded. The left panels show the best-fitting model not including depth: the correspondence between these fits and those of the full analysis (Supplementary Fig. 5) indicate the extent to which this subset of observations is representative of the full analysis. The right panels show the best-fitting model including depth as a covariate. For visualization of the basin and interannual variability in mortality thresholds, these latter thresholds represent predicted mortality probabilities at the dataset's median depth of 6.7m. The vertical axis is the probability of moderate or greater coral mortality calculated from the subset of the bleaching/mortality database that contained maximum depth values (n = 5,547 surveys in AP; 5,158 in CA; and 1,347 in IM). Curves extend across the domain of DHW values apparent for that basin and year. Solid lines represent the mean probability of bleaching, and shaded regions represent 95% confidence intervals on the mean. The data and code to generate this figure are provided in Zenodo (https://doi.org/10.5281/zenodo.15114357).

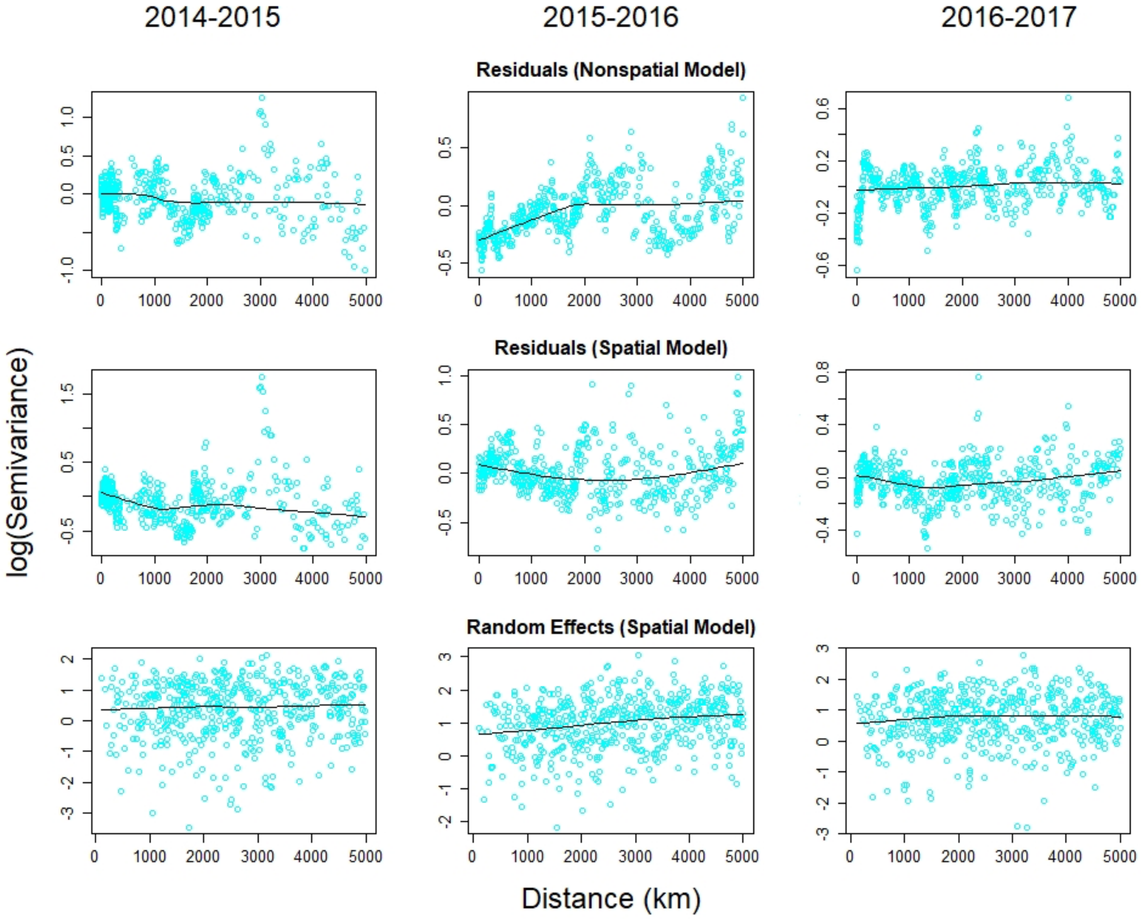

Supplementary Figure 10. Semivariograms of residuals of fixed-effect only models (top row), residuals of model including random effect of Regional Virtual Station (middle row), and random effect estimates from the model including random effects of Regional Virtual Station (bottom row) over the first 5,000 kms of distance, for moderate or greater bleaching (>10% of corals). Note the pronounced increase in log(semivariance) with increasing distance over the first few thousand kilometers in 2015–2016 for the fixed-effects only model (top row, middle). This yielded several million semivariance estimates. So, for plotting, these were then averaged over 500 distance bins with each bin set to average the same number of semivariances, and then log-transformed. Spatial autocorrelation over trend lines were plotted using the function "scatter.smooth" in the base R package with degree 1 and span 2/3. The data and code to generate this figure are provided in Zenodo (https://doi.org/10.5281/zenodo.15114357).

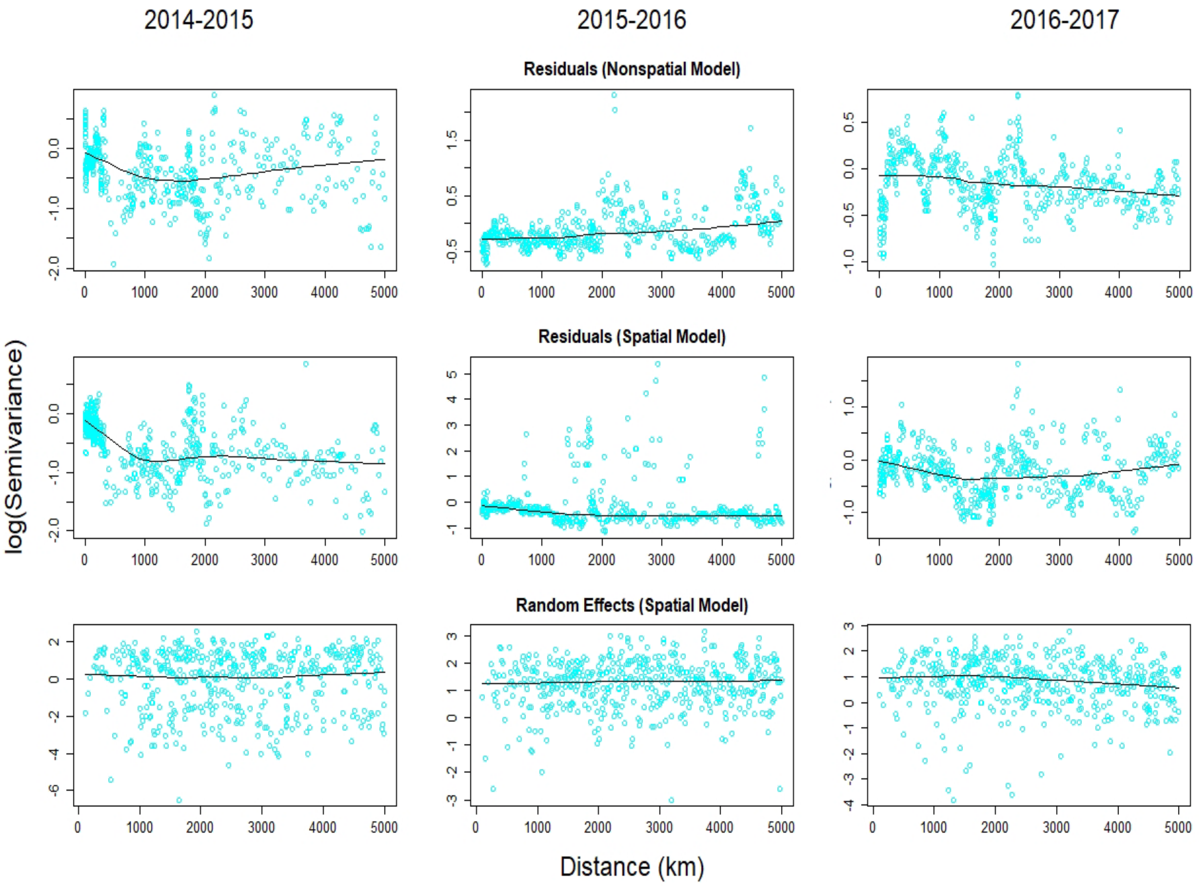

Supplementary Figure 11. Semivariograms of residuals of fixed-effect only models (top row), residuals of model including random effect of Regional Virtual Station (middle row), and random effect estimates from the model including random effects of Regional Virtual Station (bottom row) over the first 5,000 kms of distance, for severe bleaching (>50% bleached). Note the pronounced increase in log(semivariance) with increasing distance over the first few hundred kilometers in 2016–2017 for the fixed effects only model (top row, right). See Supplementary Fig. 10 for details on calculation and plotting of semivariances.

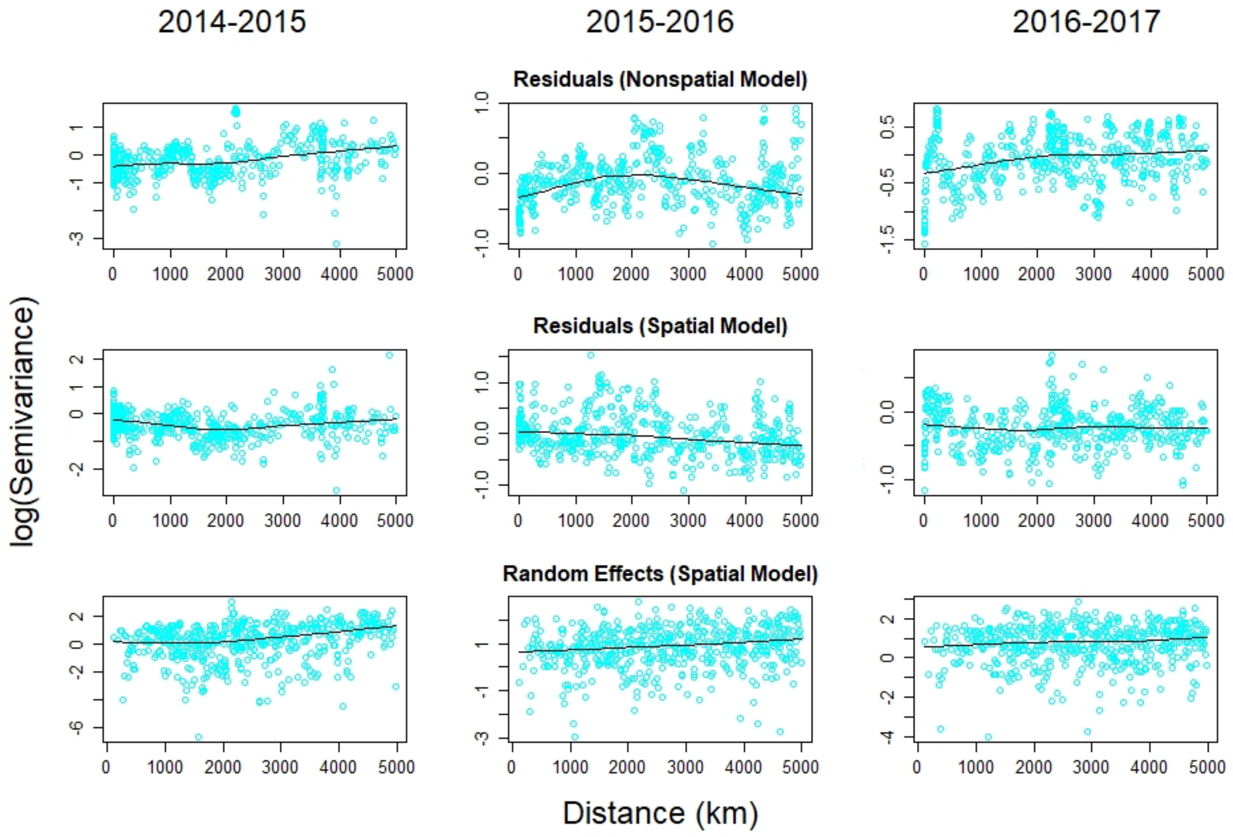

Supplementary Figure 12. Semivariograms of residuals of fixed-effect only models (top row), residuals of model including random effect of Regional Virtual Station (middle row), and random effect estimates from the model including random effects of Regional Virtual Station (bottom row) over the first 5000 kms of distance, for moderate or greater mortality (>10% of corals). See Supplementary Fig. 10 for details on calculation and plotting of semivariances.
