# Supplementary material for: Severe and widespread coral reef damage during the 2014-2017 Global Coral Bleaching Event
Source: Nat Commun. 2026 Feb 10;17:1318. doi: 10.1038/s41467-025-67506-w (PMC12891614; doi:10.1038/s41467-025-67506-w)
Supplement: Supplementary file 2 — Description of Additional Supplementary Information [file 41467_2025_67506_MOESM2_ESM.pdf]

Description of Additional Supplementary Files

File Name: Supplementary Data 1
Description: Bleaching and mortality observation database consisting of all aerial and in situ survey observation s collected over the period of June 2014 – May 2017 that delineated the Third Global Coral Bleaching Event.

The four tabs are:

1. Bleaching & Mortality Database: The first tab is the main data sheet, containing the bleaching and mortality observations.
2. Column Descriptions: The second tab gives the title of each column in the main database, and a description of the information contained in that column.
3. The third tab is a worksheet giving the two-letter codes used for all regions and countries from which the observations came.
4. The fourth tab defines all letter and number codes used in the spreadsheet (bleaching and mortality severity, data type, and quality assessment).

File Name: Supplementary Movie 1
Description: Animation of Global spatial pattern of maximum heat stress over the period of June 2014 – May 2017 that delineated the Third Global Coral Bleaching Event.
