# Supplementary figures and images for: Severe and widespread coral reef damage during the 2014-2017 Global Coral Bleaching Event
Source: Nat Commun. 2026 Feb 10;17:1318. doi: 10.1038/s41467-025-67506-w (PMC12891614; doi:10.1038/s41467-025-67506-w)

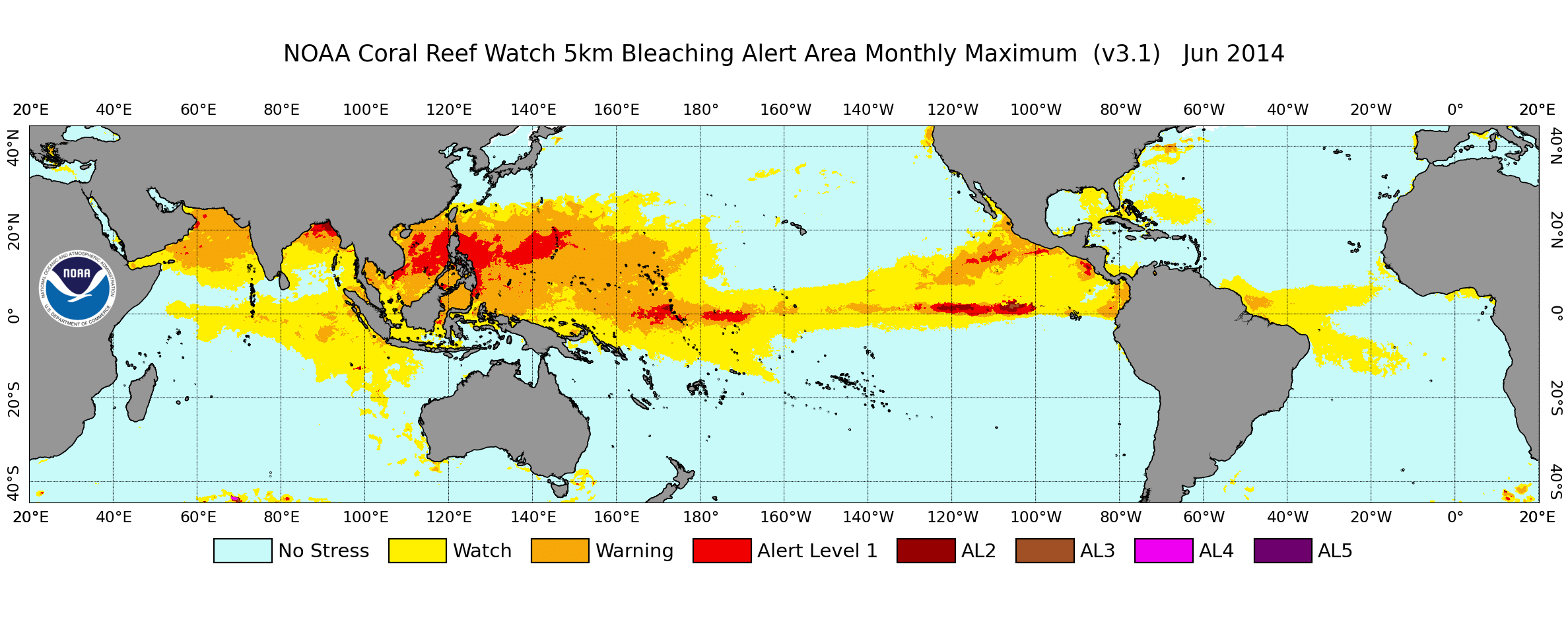

Supplement: Supplementary file 3 — Supplementary Video 1 [file 41467_2025_67506_MOESM3_ESM.gif]
