# Supplementary material for: Severe and widespread coral reef damage during the 2014-2017 Global Coral Bleaching Event
Source: Nat Commun. 2026 Feb 10;17:1318. doi: 10.1038/s41467-025-67506-w (PMC12891614; doi:10.1038/s41467-025-67506-w)
Supplement: Supplementary file 5 — Reporting Summary [file 41467_2025_67506_MOESM5_ESM.pdf]

nature portfolio

Corresponding author(s): Mark Eakin, Scott Heron, Sean Connolly

Last updated by author(s): 2025-03-24

Reporting Summary

Nature Portfolio wishes to improve the reproducibility of the work that we publish. This form provides structure for consistency and transparency in reporting. For further information on Nature Portfolio policies, see our Editorial Policies and the Editorial Policy Checklist.

Statistics

For all statistical analyses, confirm that the following items are present in the figure legend, table legend, main text, or Methods section.

n/a	Confirmed	
[ ]	[X]	The exact sample size (*n*) for each experimental group/condition, given as a discrete number and unit of measurement
[ ]	[X]	A statement on whether measurements were taken from distinct samples or whether the same sample was measured repeatedly
[ ]	[X]	The statistical test(s) used AND whether they are one- or two-sided<br>*Only common tests should be described solely by name; describe more complex techniques in the Methods section.*
[ ]	[X]	A description of all covariates tested
[X]	[ ]	A description of any assumptions or corrections, such as tests of normality and adjustment for multiple comparisons
[ ]	[X]	A full description of the statistical parameters including central tendency (e.g. means) or other basic estimates (e.g. regression coefficient) AND variation (e.g. standard deviation) or associated estimates of uncertainty (e.g. confidence intervals)
[X]	[ ]	For null hypothesis testing, the test statistic (e.g. *F*, *t*, *r*) with confidence intervals, effect sizes, degrees of freedom and *P* value noted<br>*Give P values as exact values whenever suitable.*
[X]	[ ]	For Bayesian analysis, information on the choice of priors and Markov chain Monte Carlo settings
[ ]	[X]	For hierarchical and complex designs, identification of the appropriate level for tests and full reporting of outcomes
[X]	[ ]	Estimates of effect sizes (e.g. Cohen's *d*, Pearson's *r*), indicating how they were calculated

Our web collection on statistics for biologists contains articles on many of the points above.

Software and code

Policy information about availability of computer code

Data collection	Remote sensing-derived data were extracted, calculated and plotted using IDL 8.7 and Python 3.8.5, with the following required Python computing environment:<br>netcdf4 version 1.5.5.1, scipy version 1.6.0, python version 3.8.5, matplotlib version 3.3.3, pytables version 3.6.1, basemap version 1.2.2, basemap-data-hires version 1.2.2, pandas version 1.2.3, xarray version 0.20.1, pooch version 1.4.0, scikit-learn version 1.0.2, numba version 0.55.0, pyts version 0.12.0. All code is available on Zenodo (DOI: 10.5281/zenodo.15114357, https://zenodo.org/records/15114357).
Data analysis	All bleaching analyses were conducted in R (version 4.4.2), using the libraries (glmmTMB version 1.1.10; geodist version 0.1.0; nlme version 3.1.166) and functions as specified in the main text and Extended Data. Code to regenerate all results (including MS figures), and fitted model objects (saved in Rdata files) are available on Zenodo (DOI: 10.5281/zenodo.15114357, https://zenodo.org/records/15114357).

For manuscripts utilizing custom algorithms or software that are central to the research but not yet described in published literature, software must be made available to editors and reviewers. We strongly encourage code deposition in a community repository (e.g. GitHub). See the Nature Portfolio guidelines for submitting code & software for further information.

Data

Policy information about availability of data

All manuscripts must include a data availability statement. This statement should provide the following information, where applicable:

- Accession codes, unique identifiers, or web links for publicly available datasets
- A description of any restrictions on data availability
- For clinical datasets or third party data, please ensure that the statement adheres to our policy

Research involving human participants, their data, or biological material

Policy information about studies with human participants or human data. See also policy information about sex, gender (identity/presentation), and sexual orientation and race, ethnicity and racism.

Reporting on sex and gender	N/A
Reporting on race, ethnicity, or other socially relevant groupings	N/A
Population characteristics	N/A
Recruitment	N/A
Ethics oversight	N/A

Note that full information on the approval of the study protocol must also be provided in the manuscript.

Field-specific reporting

Please select the one below that is the best fit for your research. If you are not sure, read the appropriate sections before making your selection.

☐ Life sciences ☐ Behavioural & social sciences ☒ Ecological, evolutionary & environmental sciences

For a reference copy of the document with all sections, see nature.com/documents/nr-reporting-summary-flat.pdf

Ecological, evolutionary & environmental sciences study design

All studies must disclose on these points even when the disclosure is negative.

Study description	This study is an analysis of coral bleaching and mortality data worldwide that was collected during the period of June 2014-May 2017.
Research sample	The research sample consisted of all field observational surveys of coral reef habitat that we could obtain, for which bleaching and/or mortality data were available, during the study time period. Specifically, data were either submitted directly to the researchers by individuals, or extracted from publicly available databases.
Sampling strategy	Coral Reef Watch sent out multiple calls for coral reef survey data (including observations of the absence of bleaching and/or mortality) conducted during the period 1 June 2014-31 May 2017. We sought out a wide range of potential collaborators through direct contacts, calls for data via the NOAA Coral-List listserv, published calls for data, and an appeal through the team who created the film Chasing Coral. We also extracted data on coral bleaching and mortality (where available) covering June 2014 – May 2017 from multiple regional/international databases/sources including Donner et al. 2017 (see paper for full reference), Reef Check International, Atlantic and Gulf Rapid Reef Assessment (AGRRA), Coastal Oceans Research and Development - Indian Ocean (CORDIO) East Africa, NOAA's National Coral Reef Monitoring Program (NCRMP), and Great Barrier Reef surveys.
Data collection	(1) measures of coral bleaching as coral cover bleached (%), number of coral colonies bleached (n) and total number of colonies surveyed (N), or both; and/or (2) measures of coral mortality as coral cover dead (%), number of coral colonies dead (n) and total number of colonies surveyed (N), or both; (3) observation date; (4) observation location, including latitude, longitude, and reef site name; (5) data source; and (6) survey method used. We converted percentage bleached (and mortality, where available) into categorical variables following the same protocol as ReefBase (http://www.reefbase.org).
Timing and spatial scale	Only surveys conducted within June 2014-May 2017 were included. The spatial scope was global. Individual surveys varied in extent.
Data exclusions	All data for which % bleaching could be obtained were used in bleaching analyses, and all data for which % mortality could be obtained were used in mortality analyses.
Reproducibility	N/A. This was an observational study of ecological response to a specific historical event. (Analyses and figures, however, can be reproduced using the code, data, and fitted model objects that will be made available on Zenodo prior to publication.)
Randomization	N/A. The data were observational, not based on experimental manipulation
Blinding	N/A/. The data were observational, not based on experimental manipulation

Did the study involve field work? ☐ Yes ☒ No

Reporting for specific materials, systems and methods

We require information from authors about some types of materials, experimental systems and methods used in many studies. Here, indicate whether each material, system or method listed is relevant to your study. If you are not sure if a list item applies to your research, read the appropriate section before selecting a response.

Materials & experimental systems

n/a	Involved in the study
☒	☐ Antibodies
☒	☐ Eukaryotic cell lines
☒	☐ Palaeontology and archaeology
☒	☐ Animals and other organisms
☒	☐ Clinical data
☒	☐ Dual use research of concern
☒	☐ Plants

Methods

n/a	Involved in the study
☒	☐ ChIP-seq
☒	☐ Flow cytometry
☒	☐ MRI-based neuroimaging

Plants

Seed stocks	N/A
Novel plant genotypes	N/A
Authentication	N/A
